# Molecular Detection of Bacterial Abortion Agents and Assessment of Serum MDA and GSH Levels in Aborting Small Ruminants in Siirt Province

**DOI:** 10.3390/ani16213508

**Published:** 2026-11-06

**Authors:** Muazzez Yeşilyurt, Serpil Aygörmez, Özgül Gülaydın, Mushap Kuru

**Affiliations:** 1 Department of Microbiology, Faculty of Veterinary Medicine, Siirt University, Siirt 56100, Türkiye; ozgul.gulaydin@siirt.edu.tr; 2 Department of Biochemistry, Faculty of Veterinary Medicine, Kafkas University, Kars 36000, Türkiye; serpilaygormez@hotmail.com; 3 Department Obstetrics and Gynecology, Faculty of Veterinary Medicine, Kafkas University, Kars 36000, Türkiye; mushap.kuru@kafkas.edu.tr

**Keywords:** abortion, bacteria, *Campylobacter* spp., sheep, goat, malondialdehyde, glutathione

## Abstract

Abortions in sheep and goat farming cause significant economic losses and a severe decline in productivity. The aim of this study was to detect the presence of *Chlamydia abortus*, *Coxiella burnetii*, *Listeria* spp., *Listeria monocytogenes*, and *Campylobacter* spp.—pathogens responsible for abortion cases in small ruminants—and to evaluate certain serum oxidative stress parameters. A total of 215 vaginal swab samples (sheep = 158; goats = 57) were examined using the Polymerase Chain Reaction (PCR) method. To evaluate the impact of the predominant bacterial agent, serum malondialdehyde and glutathione levels were analyzed in a focused subset (*n* = 140) comprising *Campylobacter* spp.-positive animals (*n* = 70) and pathogen-negative aborting controls (*n* = 70). PCR analysis revealed an overall positivity rate of 34.41% (*n* = 74), with *Campylobacter* spp. detected in 32.55% (70/215) and *Chlamydia abortus* in 1.86% (4/215) of samples, while no other targeted agents (*Coxiella burnetii* and *Listeria* spp.) were detected. In the *Campylobacter* spp.-positive group, serum malondialdehyde levels (32.14 ± 0.89) were significantly higher and glutathione levels (32.92 ± 3.25) were markedly lower compared to controls (9.30 ± 2.33 and 103.12 ± 1.63, respectively; *p* < 0.001; effect size r = 0.86), showing significant intra-species differences (*p* < 0.05) but no inter-species variation between infected sheep and goats (*p* > 0.05). In conclusion, *Campylobacter* spp. may play a significant role in small ruminant abortions in Siirt, which might be accompanied by cellular oxidative stress.

## Simple Summary

Pregnancy losses in sheep and goats pose significant economic challenges for livestock producers and negatively impact herd productivity. This study investigated specific bacterial agents associated with pregnancy losses and two key cellular stress markers in affected animals across small ruminant farms in Siirt Province, Türkiye. Evaluation of reproductive samples obtained from more than two hundred sheep and goats identified *Campylobacter* as the most prevalent targeted pathogen, whereas *Chlamydia* was detected in a limited number of cases. Furthermore, preliminary blood evaluations revealed a cellular imbalance characterized by elevated indicators of tissue damage and reduced levels of protective antioxidants in *Campylobacter*−positive animals. Although limited to specific parameters, these findings suggest that *Campylobacter* may play a notable role in regional pregnancy losses and could be associated with oxidative imbalance in affected animals. Consequently, these data provide useful baseline evidence to support targeted herd management and preventative strategies for local veterinarians and farmers.

## 1. Introduction

Small ruminant farming, which makes a significant contribution to the national economy, holds immense importance, particularly in the Southeastern Anatolia Region, owing to its favorable vegetation cover and geographical location. Well-adapted to Türkiye’s geographical conditions and raised for their meat, milk, and wool, small ruminants have also become a key source of livelihood in the Siirt region due to their ability to utilize marginal lands [1,2]. Additionally, small ruminant farming constitutes the most cost-effective livestock sector in Türkiye owing to favorable climate and geographical conditions [3,4]. However, abortion cases occurring in small ruminants lead to significant economic losses [5]. Abortion cases can stem from non-infectious causes—including nutritional, genetic, stress, and physical factors—as well as bacterial, viral, parasitic, or fungal agents [6,7]. Bacterial agents responsible for abortion notably include *Campylobacter* spp., *Listeria monocytogenes* (*L. monocytogenes*), *Coxiella burnetii* (*C. burnetii*), and *Chlamydia abortus* (*C. abortus*) [6,7,8,9].

*C. abortus* and *C. burnetii*, which are Gram-negative, obligate intracellular microorganisms, cause severe reproductive health problems in livestock. Furthermore, possessing a zoonotic character, these agents can also be transmitted to humans [10,11]. It has been reported that the incidence of *C. abortus* in pregnant women in the United Kingdom remains limited to an average of two cases per year, whereas *C. burnetii*-sourced infections in Southern France are detected in one out of every 540 pregnant women [12,13]. Examination of national and international studies indicates that the prevalence rates of *C. abortus* and *C. burnetii* in sheep and goats are in the ranges of 7.8–42.8% [14,15,16,17,18] and 0–14.5% [14,15,17,18], respectively. Furthermore, recent molecular and epidemiological evidence from Pakistan underscores the continued endemic presence and regional public health burden of *C. burnetii* in small ruminant livestock across Asian pastoral systems, reinforcing the necessity of routine screening [19].

*L. monocytogenes*, a motile, Gram-positive, and facultative intracellular microorganism, negatively impacts productivity in animals by causing clinical conditions such as abortion, stillbirth, septicemia, encephalitis, and keratitis [20]. Studies have reported that the prevalence of *L. monocytogenes* in sheep and goats ranges between 0 and 13.3% [5,15,18,21]. Furthermore, it can also cause symptoms such as abortion, septicemia, and meningitis in humans [22].

Thermotolerant *Campylobacter* species, recognized as a cause of gastroenteritis in both humans and animals, can continue to spread and colonize the intestines without eliciting any clinical signs [23,24]. However, in infected animals, they can also lead to abortion, weak lamb births, and mortality resulting from metritis [25]. In humans, these species can cause diarrhea, fever, weight loss, meningitis, myocarditis, and significant neurological conditions such as Miller Fisher and Guillain–Barré syndromes [23,26]. *Campylobacter* spp. can contaminate other organs and tissues during slaughter, thereby contributing to environmental contamination and dissemination. Furthermore, aborted fetuses, placentas, and the fecal–oral route serve as the primary sources of transmission [27,28]. Beyond these factors, workers in animal housing facilities and livestock processing plants [29], as well as wildlife and rodents, play a significant role in contamination and transmission [30]. In studies investigating abortion outbreaks, *Campylobacter* spp. prevalence rates ranging from 1.7% to 23.2% have been reported in evaluated sheep and goat herds, respectively [15,17,18,31].

Biochemical changes occurring in serum are generally associated with cellular damage. Cellular damage leads to alterations in specific biomarkers that can be utilized to determine tissue damage and inflammation [32]. In this context, oxidative stress parameters—resulting from the increase in reactive oxygen species (ROS) and the organism’s inability to neutralize them—serve as auxiliary markers in the diagnosis and prognosis of various infectious diseases [33,34,35]. Malondialdehyde (MDA), a natural end product of lipid peroxidation that increases during oxidative stress, is a sensitive and specific biomarker widely used to evaluate cellular oxidative damage [36,37]. Glutathione (GSH) is another key biomarker among antioxidants. Serving as a cofactor for numerous antioxidant enzymes, GSH also enables the regeneration of vitamins C and E, thereby maintaining redox homeostasis [38,39]. Because these distinct bacterial agents frequently share overlapping clinical presentations (predominantly late-term abortions), seasonal patterns, and transmission dynamics under extensive and transhumant management systems, evaluating them within a combined differential diagnostic panel is essential for targeted health control and effective public health management [5,6,7,8,9,27].

Accordingly, the primary objective of this study was to determine the molecular prevalence of key bacterial abortion agents (*Coxiella burnetii*, *Campylobacter* spp., *Chlamydia abortus*, and *Listeria* spp.) using PCR in sheep and goats raised in Siirt Province. Furthermore, while PCR screening provides essential diagnostic and epidemiological data, molecular detection alone offers limited information regarding the pathophysiological status of infected animals. To bridge this gap, serum MDA and GSH levels were evaluated in animals testing positive for whichever bacterial pathogen was identified as the most prevalent in the study group, serving as an indicator of host cellular stress responses. By integrating broader regional pathogen screening with targeted biochemical evaluations of the dominant positive cases, this study aims to contribute to both regional disease surveillance and a clearer understanding of host–pathogen interactions in natural livestock abortions.

## 2. Materials and Methods

### 2.1. Study Area and Animal Material

In this study, vaginal swab samples collected from 158 sheep and 57 goats that aborted in different small ruminant enterprises in the Siirt region and blood serum samples taken from the same animals were used.

This study was conducted between January 2026 and April 2026 in 10 small-ruminant-rearing family farms located in the province of Siirt in Southeastern Türkiye. A convenience sampling method was employed to select animals that experienced clinical abortion during the study period. A total of 215 animals (158 sheep and 57 goats) that had aborted and met the study criteria were identified; vaginal swabs and blood serum samples were collected from these animals.

Inclusion criteria consisted of female sheep or goats raised on small-ruminant farms in Siirt province that had a confirmed abortion during the second or third trimester of pregnancy and that had not received systemic antibiotic or antimicrobial treatment within the 15 days prior to sample collection.

Exclusion criteria included cases of abortion confirmed to be caused by known factors such as physical/mechanical trauma, severe stress, or non-infectious nutritional deficiencies; animals that had received systemic antibiotic treatment within the 15 days prior to sample collection; and samples that had undergone hemolysis—and were thus compromised—prior to laboratory analysis.

### 2.2. Sample Size and Statistical Power

The sample size for this study (*n* = 215; 158 sheep and 57 goats) was not determined through a prospective sample size calculation; rather, it was based on the conditions observed in the field, encompassing all clinical abortion cases reported during the study period that met the inclusion criteria. To characterize the precision and statistical sensitivity of these field-derived samples, the following supplementary calculations were performed:

#### 2.2.1. Epidemiological Precision

Under a simple random sampling assumption, standard epidemiological formulas for proportion estimation [40] indicate that a sample size of *n* = 215, assuming *p* = 0.50, corresponds to a nominal maximum margin of error of approximately ±6.7 percentage points at the 95% confidence level. Because the study sample was obtained by convenience sampling, this value is reported as an approximate precision benchmark rather than a design-based population margin of error.

#### 2.2.2. Biochemical Power

The biochemical comparisons were based on a subset of 140 animals, comprising 70 *Campylobacter* spp.-positive animals and 70 pathogen-negative controls. A sensitivity-based power calculation using Cohen’s standardized effect-size framework [41] indicated that 70 animals per group provide approximately 83.6% power to detect a standardized effect size of Cohen’s d = 0.50 at a two-sided α = 0.05. This calculation was used as a supplementary assessment of the statistical sensitivity of the biochemical comparison.

### 2.3. Sample Collection and Storage Conditions

#### 2.3.1. Vaginal Swab Samples

Vaginal swab samples were collected from aborting sheep and goats. The collected swab samples were placed into Stuart transport medium and transported to the Laboratory of the Department of Microbiology, Faculty of Veterinary Medicine, Siirt University, under cold chain conditions within a short time. The collected swab samples were stored at −20 °C until DNA isolation.

#### 2.3.2. Blood Samples

Blood samples were collected from the jugular vein into vacuum tubes without anticoagulant. The blood samples brought to the laboratories of the Department of Microbiology, Siirt University, were centrifuged at 10,000× *g* for 10 min and stored at −20 °C to be used in oxidative stress analyses. To maintain biochemical integrity, storage duration was kept under four months, and samples were subjected to a single standardized freeze–thaw cycle prior to oxidative stress measurements. Sample collection, transport, and processing protocols were standardized across all study groups.

Vaginal swab samples and corresponding blood samples were collected concurrently from aborting animals within 24 to 48 h following the abortion event. This timeline was strictly maintained to minimize environmental contamination, maximize the likelihood of detecting shed bacterial DNA, and ensure that serum oxidative stress indicators accurately reflected the acute physiological response associated with the abortion.

### 2.4. DNA Extraction

Genomic DNA from vaginal swab samples was obtained using a commercial kit (K0722, Thermo Fisher, Waltham, MA, USA). The obtained DNA samples were stored at −20 °C until used in PCR analysis.

### 2.5. PCR Amplification

A commercial master mix (EcoTaq 2x PCR Master Mix, ET-1, EcoTech Biotechnology, Erzurum, Türkiye) was used in the preparation of the PCR mixture, and PCR optimization was performed in accordance with the manufacturer’s recommendations. For the purpose of optimizing the mixture, 5 µL of genomic DNA, 1.5 µL of each primer (Forward and Reverse individually, 10 µM stock concentration), and 4.5 µL of PCR water were added to 12.5 µL of master mix, bringing the total volume to 25 µL. Thermal cycling was performed using the following general framework: an initial denaturation at 94 °C for 10 min, followed by 35 cycles of denaturation at 94 °C, target-specific annealing (56 °C to 60 °C), and extension at 72 °C (specific cycle durations for each target pathogen are detailed in Table 1, with a final extension at 72 °C for 10 min. The amplicons obtained from PCR were subjected to 1.5% agarose gel electrophoresis and visualized using a gel imaging device (Gene-Box FX, ER Biotech, Ankara, Türkiye). A sample was defined as positive if a single, distinct band corresponding to the expected target amplicon size (Table 1) was observed in comparison with a standard DNA ladder, provided that no amplification was detected in the non-template negative control.

Quality control during molecular testing was routinely maintained by including a non-template negative control (nuclease-free water) in every PCR run to monitor for cross-contamination. Commercial positive control DNA strains and target sequencing verification were not routinely conducted; therefore, pathogen identification relied strictly on target-specific primer amplification, expected amplicon length verification via standard DNA ladder, and negative control validation.

### 2.6. Determination of Oxidative Stress Parameters

In order to evaluate oxidative stress in blood serum samples, MDA and GSH levels were analyzed using spectrophotometric methods. Serum samples were stored at −20 °C until biochemical analyses were performed. All analyses were completed within a single day.

#### 2.6.1. Determination of MDA Levels

MDA levels were determined according to the method described by Placer et al. [46]. The absorbance of the pink-colored complex formed by the reaction of MDA, an aldehyde end product of lipid peroxidation, with thiobarbituric acid (TBA) was measured at a wavelength of 532 nm (Bio-Tek Gen5 Epoch, Santa Clara, CA, USA). 1,1,3,3-Tetraethoxypropane (purity ≥ 96%, CAS No: 122-31-6, Sigma-Aldrich, St. Louis, MO, USA) was used to generate the MDA standard curve.

#### 2.6.2. Determination of GSH Levels

The method used by Sedlak and Lindsay was used for the measurement of GSH levels [47]. The method is based on the principle of the reduction of the 5,5′-dithiobis (2-nitrobenzoic acid) (DTNB) compound in the reagent by sulfhydryl (-SH) groups in the medium. The intensity of the chromophoric yellow color formed as a result of the reduction was recorded spectrophotometrically at a wavelength of 412 nm in a spectrophotometer (Bio-Tek Gen5 Epoch, Santa Clara, CA, USA). Glutathione (purity ≥ 98.0%, CAS No: 70-18-8; Sigma-Aldrich, St. Louis, MO, USA) was used to generate the GSH standard curve.

To prevent analytical bias during laboratory processing, laboratory personnel performing the biochemical assays were blinded to the PCR status of the corresponding animal samples until all biochemical data were recorded.

### 2.7. Statistical Analysis

Statistical analyses of the biochemical data obtained in the study were performed using Python software (Version 3.11; Python Software Foundation, Wilmington, DE, USA); SciPy (v1.11), Statsmodels (v0.14), Pandas (v2.1), and Seaborn (v0.13) libraries were utilized during the analysis and visualization processes. The conformity of data belonging to continuous variables (MDA and GSH) to the theoretical normal distribution was evaluated by the Shapiro–Wilk test, skewness and kurtosis coefficients, and examination of Q-Q plots. Since the variables were found not to show a normal distribution (*p* < 0.05), non-parametric methods were preferred in the analyses. Descriptive statistics are presented as median (interquartile range, IQR: Q3-Q1) and mean ± standard deviation (SD).

The Mann–Whitney U test was applied to compare serum MDA and GSH levels according to *Campylobacter* spp. infection status (positive and negative) and animal species (sheep and goat). To control the family-wise error rate across multiple subgroup comparisons, a Bonferroni adjustment was implemented, setting the significance threshold to alpha_adjusted = 0.05/5 = 0.01. To evaluate the clinical and biochemical significance of differences between groups, the non-parametric effect size coefficient (r = |Z|/√N) was calculated (Z: standardized Z score, N: total sample size). Effect sizes were classified as negligible for r < 0.10, small for 0.10 ≤ r < 0.30, medium for 0.30 ≤ r < 0.50, and large for r ≥ 0.50. The 95% confidence intervals (95% CIs) of the differences between medians were determined using the percentile Bootstrap method with 5000 resamples.

The relationship between serum MDA and GSH levels was evaluated separately in the overall sample (*n* = 140) and in subgroups (based on infection status and species) using Spearman rank correlation analysis (ρ). Scatter plots were created to visualize spatial clustering patterns between groups. The baseline statistical significance level was set at *p* < 0.05 for all analyses.

## 3. Results

### 3.1. PCR Analysis Results

**Figure 1:**
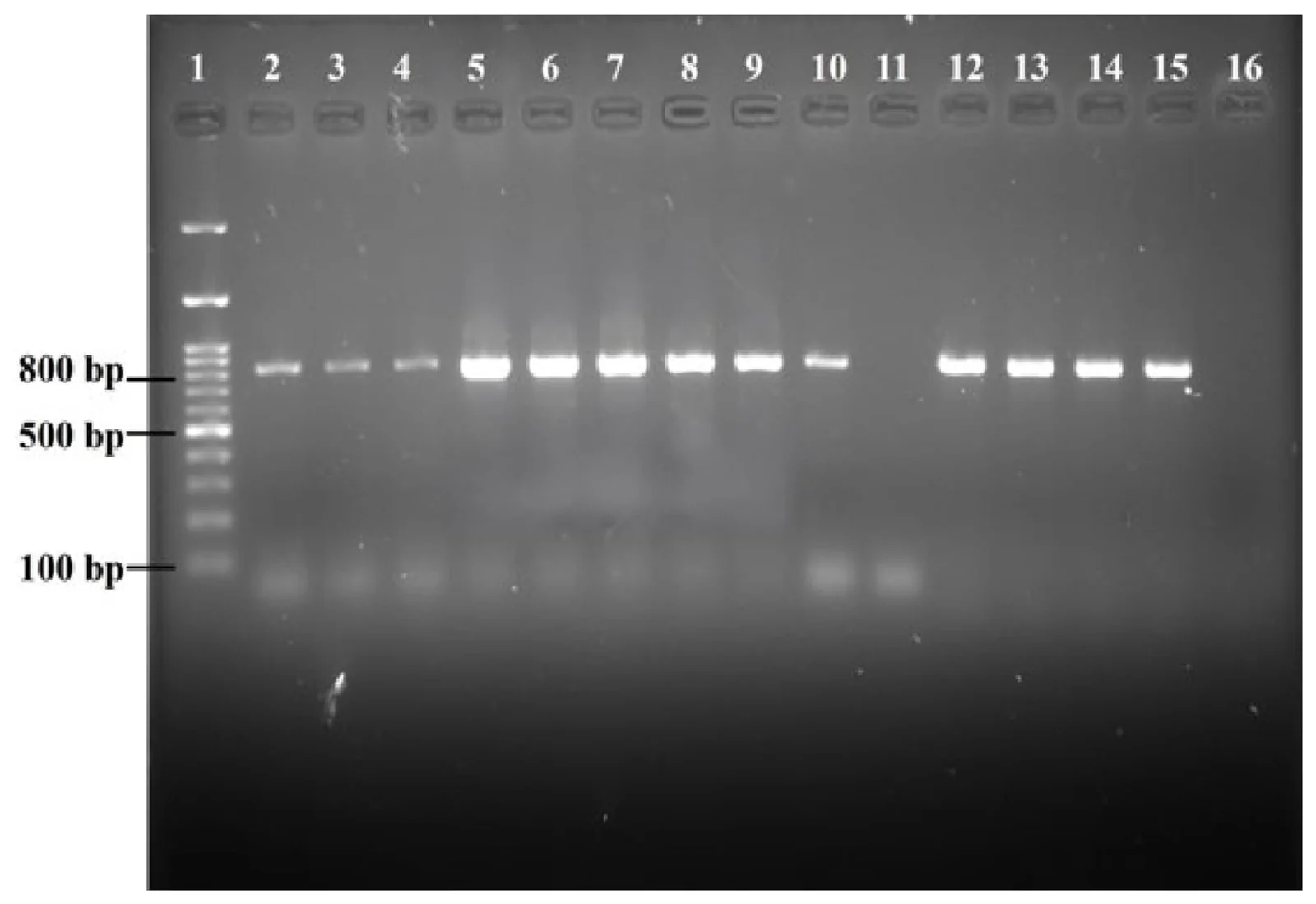
Agarose gel electrophoresis image of PCR amplicons [1: 100 bp DNA Ladder; 2–10: *Campylobacter* spp. positive isolates (816 bp); 11: *Campylobacter* spp. negative control; 12–15: *C. abortus* positive isolates (821 bp); 16: *C. abortus* negative control].

**Figure 2:**
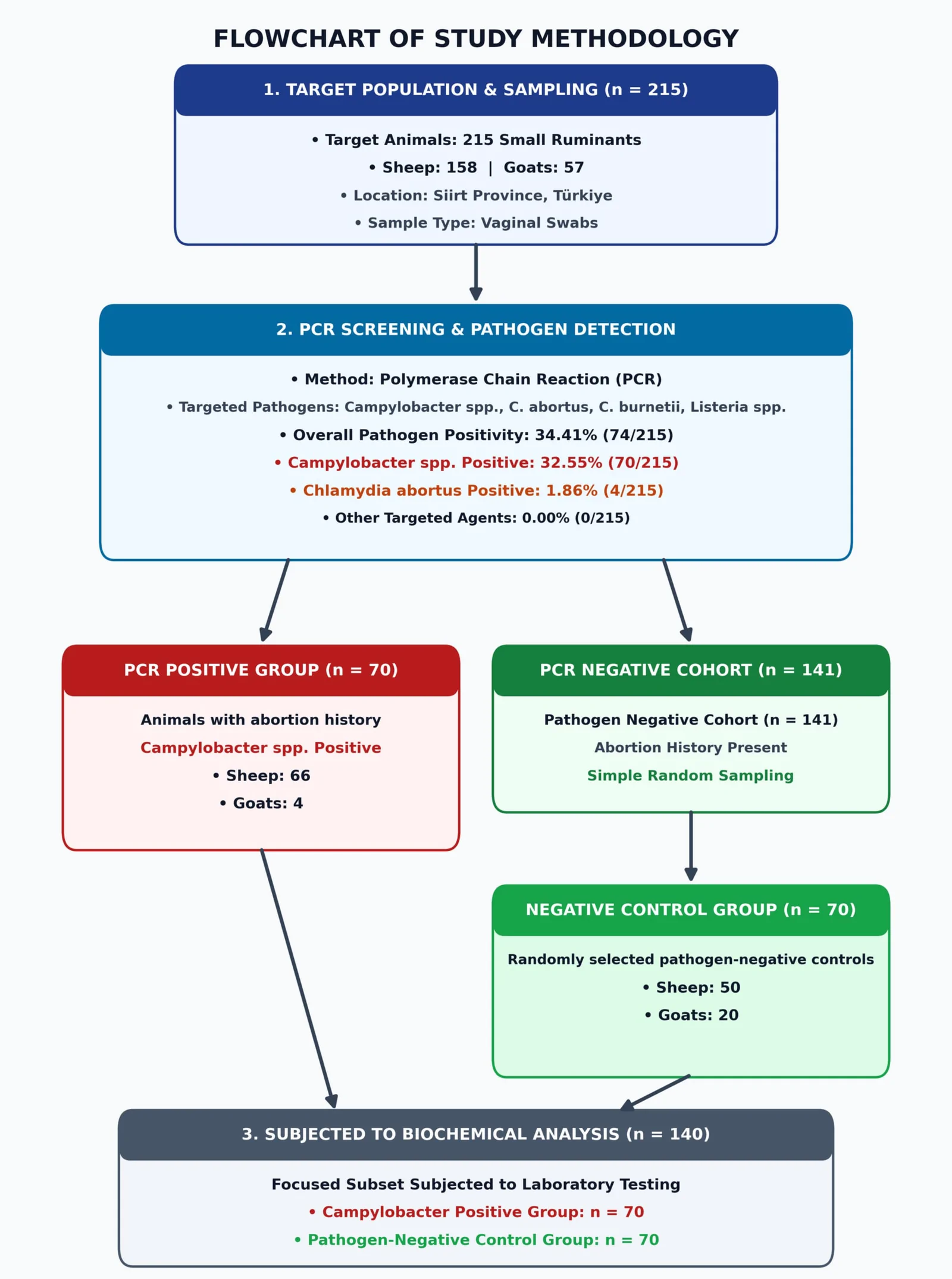
Flow diagram illustrating the study population, PCR screening outcomes for *Campylobacter* spp., simple random sampling process for the control group, and final group allocations. All evaluated animals included in the study had a history of abortion.

In this study, bacterial agents were detected in 74 (34.41%, 95% CI: 28.39–40.99%) of the 215 (sheep = 158; goats = 57) samples analyzed by PCR. Of the 215 samples examined, 70 (32.55%, 95% CI: 26.65–39.08%) were identified as *Campylobacter* spp. and 4 (1.86%, 95% CI: 0.73–4.69%) as *C. abortus*. *Campylobacter* spp. positivity was detected in 4 of the 57 goat samples (7.01%, 95% CI: 2.76–16.70%) and in 66 of the 158 sheep samples (41.77%, 95% CI: 34.37–49.57%). The samples detected positive for *C. abortus* were obtained from vaginal swab samples of 3 sheep and 1 goat (Figure 1). However, as a result of the PCR analysis performed, no positivity (0.00%, 95% CI: 0.00–1.71%) was detected for *Listeria* spp. and *C. burnetii*. A control group consisting of 70 animals (50 sheep and 20 goats) was selected from samples that tested negative for all targeted bacterial pathogens *Campylobacter* spp. by PCR, using a simple random sampling method (*Campylobacter* spp., *C. abortus*, *C. burnetii*, and Listeria spp.) via PCR. No co-infections were detected among any of the pathogen-positive animals in the study. The overall sampling workflow and the process of categorization into groups are illustrated in Figure 2.

### 3.2. Evaluation of Oxidative Stress Parameters and Pairwise Comparisons

To ensure study homogeneity and prevent the confounding effects of cross-infections on oxidative stress parameters, only serum samples confirmed to be *Campylobacter*−positive or *Campylobacter*−negative via PCR were included in the study. Four samples that tested positive for *C. abortus* via PCR were excluded from biochemical analyses, both due to an insufficient sample size for statistical evaluation and to eliminate the risk of contaminating the oxidative stress profiles of the study groups. Furthermore, these animals were not eligible for the pathogen−negative control group.

Pairwise comparisons, test statistics, effect sizes, and 95% confidence intervals for median differences regarding serum MDA and GSH levels according to *Campylobacter* spp. infection status and animal species in aborted small ruminants are presented in Table 2.

In the evaluation conducted at the overall sample level (*n* = 140), serum MDA levels of animals in the *Campylobacter* spp. positive group (median: 31.83 µmol/L) were found to be statistically significantly higher compared to the negative group (median: 9.08 µmol/L) (U = 4900.00, Z = 10.21, *p* < 0.001, r = 0.86, median difference 95% CI: [22.41–23.12]). In contrast, serum GSH levels were found to be significantly lower in the positive group (median: 33.37 µmol/L) compared to the negative group (median: 103.30 µmol/L) (U = 0.00, Z = −10.21, *p* < 0.001, r = 0.86, median difference 95% CI: [−70.60–−69.40]).

In intra-species subgroup analyses, *Campylobacter* spp. positivity led to a marked increase in serum MDA levels both in sheep (U = 3300.00, Z = 9.20, *p* < 0.001, r = 0.85) and in goats (U = 80.00, Z = 3.10, *p* = 0.002, r = 0.63). Similarly, intra-species serum GSH concentrations were statistically significantly lower in positive sheep (U = 0.00, Z = −9.20, *p* < 0.001, r = 0.85) and positive goats (U = 0.00, Z = −3.10, *p* = 0.002, r = 0.63) compared to their respective negative control groups.

In inter-species comparisons, no statistically significant difference was detected in terms of serum oxidative stress parameters between *Campylobacter* spp. positive sheep and goats (for MDA, *p* = 0.839, r = 0.03; for GSH, *p* = 0.879, r = 0.02) or between *Campylobacter* spp. negative sheep and goats (for MDA, *p* = 0.339, r = 0.11; for GSH, *p* = 0.866, r = 0.02) (Table 2).

### 3.3. Correlation and Distribution Analysis

**Figure 3:**
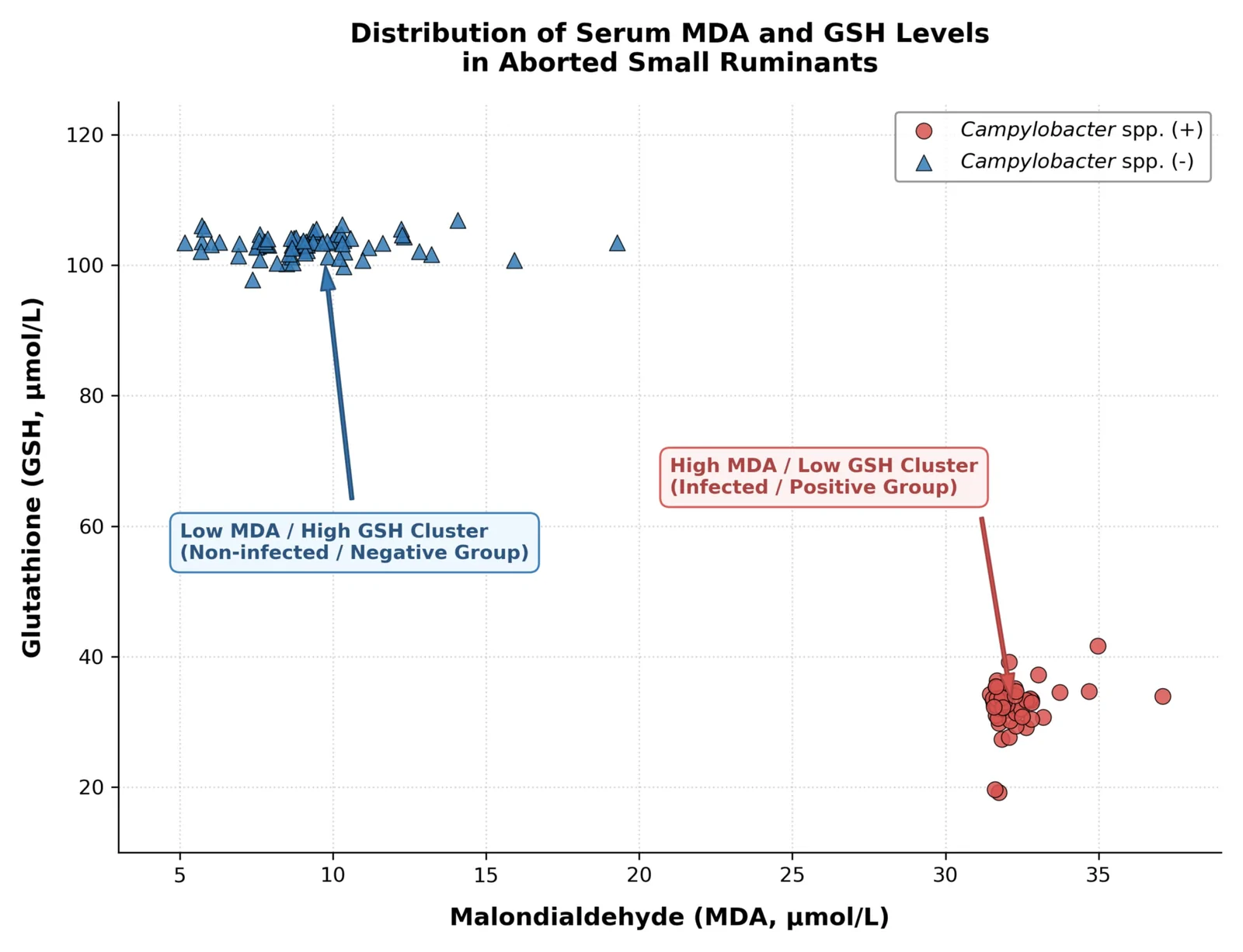
Scatter plot displaying the spatial clustering of serum MDA and GSH levels in aborted small ruminants (*n* = 140), highlighting two distinct non-overlapping clusters corresponding to *Campylobacter* spp.−positive animals (high MDA/low GSH) and pathogen−negative controls (low MDA/high GSH).

The Spearman rank correlation results regarding the relationship between serum MDA and GSH levels are presented in Table 3, and the distribution and clustering pattern of the data are presented in Figure 3.

A high-level negative correlation was detected between serum MDA and GSH levels in the entire general sample set (*n* = 140) (ρ = −0.736, *p* < 0.001). While a similar negative correlation was observed in sheep (ρ = −0.738, *p* < 0.001), this relationship was not found to be statistically significant in goats (ρ = −0.334, *p* = 0.111).

Within the *Campylobacter* spp.−positive (ρ = 0.011, *p* = 0.929) and pathogen−negative (ρ = 0.106, *p* = 0.383) subgroups themselves, no statistically significant correlation was detected between MDA and GSH parameters (Table 3).

As illustrated in Figure 3, serum biomarker values demonstrated spatial clustering into two distinct non-overlapping cohorts corresponding to *Campylobacter* spp.-positive animals (high MDA/low GSH) and pathogen-negative controls (low MDA/high GSH). The strong overall negative correlation observed across the pooled sample (ρ = −0.736, *p* < 0.001) reflects an aggregation effect between these distinct clinical clusters rather than a continuous intra-group biological correlation.

## 4. Discussion

Abortion cases observed in small ruminants constitute one of the main factors negatively affecting animal production and herd productivity. This condition generally originates from pathogens of infectious origin [48]. In Türkiye, the exact prevalence of common zoonotic abortion agents remains uncertain, primarily because the diagnosis of abortion-causing agents in small ruminants frequently relies on serological methods, and the number of studies conducted on this topic is limited [49].

Based on the reviews conducted, it was found that various studies have been carried out on the subject at both national and international levels. Within the scope of a study conducted in Iran, researchers reported detecting *Campylobacter* spp. in 3.7% (20/544), *C. abortus* in 12.3% (67/544), *C. burnetii* in 4.8% (26/544), and *Listeria* spp. in 1.3% (7/544) of the fetus/tissue samples obtained from sheep [15]. Akca et al. reported that they detected *Listeria* spp. in 8 (1.48%) of 263 vaginal swabs, 229 milk samples, and 46 aborted fetal stomach content samples taken from sheep in the Kars region of Türkiye [21]. In another study conducted in Iran, researchers detected *C. abortus* at rates of 22.8% and 20.3% in 70 sheep and 64 goat vaginal swab samples, respectively [16]. Gülaydın et al. reported that, in their study conducted in the Van region of Türkiye, they detected *Campylobacter* spp. in 2.8%, *C. abortus* in 42.8%, *C. burnetii* in 11.4%, and *L. monocytogenes* in 2.8% of various tissue and swab samples (stomach contents = 101; liver = 99; vaginal swab = 4; placenta = 4) obtained from sheep [5]. In a study conducted in Pakistan, researchers found the prevalence rates of *C. abortus* and *C. burnetii* to be 3.3% and 12.6% in 150 sheep, and 4.6% and 13.1% in 235 goats, respectively, based on blood serum samples obtained from animals with a history of abortion [50]. Şevik, in their study examining 1114 sheep and 82 goat fetus samples in Türkiye, reported the presence rates of *C. abortus*, *C. burnetii*, and *L. monocytogenes* in sheep and goat samples as 23.07–14.63%, 2.8–8.5%, and 2.4–2.4%, respectively [51]. Similarly, Yeni et al. examined vaginal swab (*n* = 31) and fetal stomach content (*n* = 25) samples obtained from sheep with a history of abortion in Türkiye using molecular methods. As a result of the research, *C. fetus* was detected at a rate of 16% in fetal stomach contents, while no positivity was encountered in vaginal swab samples and *Listeria* spp. analyses (0%) [49]. In their study, Esmaeili et al. found the presence rates of *Campylobacter* spp., *C. abortus*, *C. burnetii*, and *Listeria* spp. in 133 sheep fetus samples to be 1.7%, 25.6%, 14.5%, and 0%, respectively [18].

In this study conducted in the Siirt region, as a result of the molecular analysis of vaginal swab samples taken from sheep and goats with a history of abortion, the presence of *Campylobacter* spp. at a rate of 32.55% and *C. abortus* at a rate of 1.86% was detected. In addition, as a result of the PCR analysis, no positivity was observed for *Listeria* spp. and *C. burnetii*.

When the obtained data are compared with the results of other studies in the literature, the *Campylobacter* spp. rate we detected in the Siirt region (32.55%) is remarkably higher than the data reported in Türkiye (2.8%) [5], as well as the values obtained from international studies (3.7%; 1.7%) [15,18]. This high prevalence observed in the Siirt region may be conceptually linked to local small ruminant management practices, which predominantly rely on a transhumance (highland/winter pasture) system. Under these conditions, flocks experience transport stress and frequently share water and pasture points. While direct epidemiological metrics (such as water sampling or seasonal movement tracking) were not systematically recorded in this study, it is plausible that challenges in maintaining herd hygiene and regular veterinary care during nomadic movement could facilitate the transmission of *Campylobacter* species via fecal–oral and genital routes. Furthermore, the prolonged shedding of the pathogen in the vaginal mucosa suggests that utilizing vaginal swabs as the primary clinical sample may have enhanced diagnostic sensitivity in detecting shed bacterial DNA. Since the PCR assay in the present study was designed for genus-level detection (*Campylobacter* spp.), species-specific differentiation (such as *C. fetus*) was not conducted. Consequently, all epidemiological and biochemical observations in this study reflect total *Campylobacter* spp. presence rather than species-specific distributions.

The 1.86% *C. abortus* detection rate observed in our study was substantially lower than those reported in previous studies in Türkiye [5 (42.8%), 49 (16%), 51 (23.07%)] and international literature [15 (12.3%), 16 (22.8%), 18 (25.6%)]; however, it was found to be at similar levels to the seroprevalence reported in sheep serum samples in Pakistan [50 (3.3%)]. It is thought that the primary methodological reason for this situation may depend on the type of sample used. In most studies reporting high prevalence in the literature, the target tissues of the agent—such as placenta, fetal liver, or fetal stomach contents—were examined. Since *C. abortus* exhibits a marked placental tropism, the duration of shedding of the agent in the vaginal canal after abortion is limited [52,53]; therefore, the fact that only vaginal swabs were used instead of tissues confirms the notion that this may have lowered the detection rate. Additionally, it is thought that the antibiotic applications empirically resorted to by nomad breeders in abortion cases also suppress vaginal shedding.

The absence of positivity for *Listeria* spp. and *C. burnetii* in the vaginal swab samples we examined shows parallelism with some studies conducted in Türkiye and abroad that report negativity for Listeria spp. (0%) [18,49], while differing from the positivities reported for *C. burnetii* in Türkiye (11.4%) [5] and Pakistan (4.6%) seroprevalence [50]. Although various national studies, such as Akca et al. [21], have reported the presence of *Listeria* species in small ruminant abortions in eastern Türkiye (detecting 1.48% overall positivity across various diagnostic samples), our zero-positivity findings suggest that *Listeria* may act as a sporadic or farm-specific opportunistic pathogen rather than a primary endemic driver of abortion outbreaks in Siirt Province. In contrast, *C. abortus* was detected at a low but definitive molecular prevalence of 1.86% (4/215) in our study cohort. Comparing these two bacterial agents highlights fundamentally distinct epidemiological patterns in pastoral production systems: while *C. abortus* is an obligate intracellular pathogen characterized by persistent endemic circulation and heavy placental shedding during late-term epizootic abortion, *Listeria* infections are typically associated with environmental contamination, silage consumption, and sporadic feedborne ingestion [20,21]. It was concluded that the *Listeria* spp. negativity in our cohort may be specifically related to the absence of silage and fermented feed usage, as well as the reliance on natural pastures in nomadic livestock farming in Siirt. The complete absence of *Listeria* spp. alongside the presence of *C. abortus* and high dominance of *Campylobacter* spp. further reinforces that small ruminant abortion etiology in extensive transhumant systems is highly variable and heavily influenced by regional management practices, environmental exposures, and host susceptibility factors. As for *C. burnetii* negativity, it suggests that this may be due to the periodic decrease in the vaginal shedding of the agent or regional differences in tick control and ectoparasite density.

Although infectious agents play an important role in the pathophysiology of abortion, the immune response induced by the pathogen in gestational tissues, tissue damage, and systemic oxidative stress are evaluated as critical intermediary mechanisms in the termination of pregnancy. This indicates that one of the potential cellular pathways associated with bacterial pregnancy losses and reproductive disorders in ruminants is systemic oxidative stress triggered during the immune response [54]. During the infection process, endotoxins and cellular components secreted by pathogens induce macrophage and neutrophil activation, leading to the release of reactive oxygen species into the environment. This situation is associated with lipid peroxidation of cellular membranes in fetoplacental tissues and systemic circulation [55]. Lipids are highly sensitive to oxidation; accordingly, lipid peroxidation products such as MDA function as potential biomarkers for the evaluation of oxidative stress. Oxidative stress, which leads to cellular damage, is generally evaluated by measuring various oxidant and antioxidant parameters [56]. Previous studies have reported that bacterial abortion agents in sheep are associated with an increase in oxidative stress indices such as MDA alongside suppression of cellular antioxidant defenses [57]. Similarly, the depletion of endogenous antioxidant reserves due to the excessive production of reactive oxygen species may compromise the host’s antioxidant defense mechanism and exacerbate tissue damage [54]. In the present study, the elevation observed in MDA levels alongside decreased GSH levels in the *Campylobacter* spp.-positive group appears generally consistent with the existing literature reporting potential shifts in cellular antioxidant balance during bacterial reproductive infections. In this study, serum MDA values of aborting sheep and goats testing positive for *Campylobacter* spp. showed a statistically significant increase compared to the pathogen-negative group. Although the calculated effect size indicates a distinct statistical separation between the groups, these cross-sectional observations reflect a strong association rather than a confirmed direct causal mechanism. Parallel to this trend in lipid peroxidation markers, lower serum GSH levels were observed in the *Campylobacter* spp.-positive group. Regarding inter-species evaluations, when *Campylobacter* spp.-positive or pathogen-negative sheep and goats were compared among themselves, no statistically significant difference was observed between the species, and the effect sizes remained negligible or small. However, these inter-species subgroup comparisons should be interpreted with caution due to the low number of infected goats in the cohort.

Furthermore, although the high effect size (r = 0.86) and distinctly divergent parameter ranges observed between the *Campylobacter* spp.-positive and pathogen-negative groups might initially suggest an analytical batch effect, the fact that the analyses were performed under standardized conditions and in accordance with blinding principles mitigates this possibility. This distinct separation suggests that pathogen presence under field conditions appears to be associated with pronounced shifts in host systemic redox status.

## 5. Conclusions

This study provides regional molecular diagnostic data indicating that *Campylobacter* spp. represents a frequently detected bacterial pathogen among aborting sheep and goats under nomadic livestock farming conditions in Siirt Province. The use of shared pasture and water sources in the region, alongside limitations in preventive veterinary protocols, creates favorable conditions for the transmission of infectious abortion agents. The zoonotic nature of the evaluated bacterial pathogens poses a public health risk for livestock breeders and field veterinarians in contact with infected animals or contaminated materials. Accordingly, enhancing biosecurity practices, improving water sanitation along transhumant routes, and increasing field awareness are essential measures. Furthermore, serum biomarker evaluations indicate that *Campylobacter* spp. positivity is associated with elevated lipid peroxidation indicators (MDA) and reduced antioxidant defenses (GSH). Within the scope of herd management practices, incorporating antioxidant combinations (such as vitamin E, selenium, and vitamin C) alongside standard antimicrobial treatments during high-risk pregnancy periods represents a clinical hypothesis that warrants further investigation in prospective interventional trials.

## 6. Study Limitations

This study has several methodological and epidemiological limitations. The preference for vaginal swab samples as a practical approach under field conditions may have limited the detection of agents with high placental tropism, such as *C. abortus*. Due to the limited number of positive goats (*n* = 4) resulting from the natural prevalence in the region, subgroup data for goats should be considered preliminary. Furthermore, due to the low natural prevalence and limited sample size of *C. abortus*-positive animals (*n* = 4), separate inferential statistical evaluation of oxidative stress biomarkers specifically for *C. abortus* infection was not feasible, restricting our biochemical observations exclusively to *Campylobacter* spp.-positive cases. Finally, although a clear cellular redox imbalance was demonstrated using MDA and GSH, the incorporation of enzymatic antioxidants (SOD, GPx) and total status indicators (TAS/TOS) into future studies will further elucidate the pathophysiological picture.

A fundamental limitation stemming from the cross-sectional design of this study is its inability to definitively establish a temporal causal relationship between bacterial infection and cellular oxidative stress. Although our findings demonstrate a strong association between the presence of *Campylobacter* spp. and marked alterations in MDA and GSH levels, it cannot be clearly distinguished whether a predisposing baseline oxidative imbalance in the host predisposed to abortion or whether acute pathogen proliferation directly triggered the observed cellular damage. To fully elucidate this temporal sequence, prospective cohort studies tracking pre- and post-infection oxidative dynamics are warranted.

Another fundamental limitation is the absence of a completely healthy control group and the consequent lack of baseline comparative blood parameters. During the field phase, operational constraints were encountered, including challenges in obtaining consent from livestock owners for invasive blood sampling from asymptomatic/healthy animals, as well as local ethics committee procedures regarding unnecessary sampling in clinically non-symptomatic groups. Consequently, the control group was restricted to animals that tested negative for the target pathogen(s) but had a history of abortion. Furthermore, other major endemic abortive pathogens affecting small ruminants—most notably *Brucella melitensis*, *Salmonella* spp., *Toxoplasma gondii*, and pestiviruses—were not diagnostically evaluated. Consequently, although the control group tested negative for *Campylobacter* spp., these animals may have been infected with other unconfirmed abortifacient agents, which could potentially influence baseline biochemical parameters. Additionally, because animals were sampled across multiple family farms, potential within-herd clustering and unmeasured environmental or nutritional confounders could not be fully adjusted for in multivariable models.

Additionally, conventional bacterial culture and primary isolation were not performed prior to molecular detection. While bacterial culture remains the traditional standard for viability assessment, it was unfeasible in this field study due to severe biosafety constraints associated with culturing intracellular zoonotic agents (*C. burnetii* and *C. abortus*) and the risk of fastidious bacterial viability loss during field transport.

Finally, limitations include the lack of DNA sequencing for representative amplicons and the absence of validated positive reference controls during PCR runs. Although assay specificity was ensured through target-specific primer design, precise band-size verification against standard DNA markers, and consistent negative controls, future studies incorporating sequence verification are recommended to further confirm strain lineages. Furthermore, since the PCR assay in the present study was designed for genus-level detection (*Campylobacter* spp.), species-specific differentiation (such as *C. fetus*) was not conducted. Consequently, all epidemiological and biochemical observations in this study reflect total *Campylobacter* spp. presence rather than species-specific distributions.

## Figures and Tables

**Table 1:** Primer sequences and amplification conditions used in the PCR identification of clinical swab samples.

Bacteria	Oligonucleotide (5′–3′)	Amplicon Size (bp)	Amplification Protocol	Reference
*Campylobacter* spp.	F: GTTAAATAGTGTTCTTGGAG	816	94 °C-60 s/56 °C-60 s/72 °C-60 s/35 cycles	[42]
	R: CTAAGATATGGCTCTAACAA			
*Listeria* spp.	F: GCTGAAGAGATTGCGAAGAAG	370	94 °C-30 s/56 °C-45 s/72 °C-30 s/35 cycles	[43]
	R: CAAAGAAACCTTGGATTTGCGG			
*Listeria* *monocytogenes*	F: GCATCTGCATTCAATAAAGA	174	94 °C-30 s/57 °C-45 s/72 °C-30 s/35 cycles	[44]
	R: TGTCACTGCATCTCCGTGGT			
*Coxiella* *burnetii*	F: TATGTATCCACCGTAGCCAGT	687	94 °C-60 s/58 °C-60 s/72 °C-60 s/35 cycles	[45]
	R: CCCAACAACACCTCCTTATTC			
*Chlamydia* *abortus*	F: CTCACCATTGTCTCAGGTGGA	821	94 °C/60 s 60 °C/60 s 72 °C/60 s 35 cycles	[45]
	R: GGCAATCAGGTGCGACAATCT			

**Table 2:** Pairwise comparisons, test statistics, and effect sizes of serum MDA and GSH levels in aborted sheep and goats according to *Campylobacter* spp. status and species.

Comparison Level	Parameter	Group	*n*	Mean ± SD	Median (IQR)	Mann–Whitney U	Z Statistic	*p*-Value	Effect Size (r)	Median Difference 95% CI
General *Campylobacter*	MDA	Positive	70	32.14 ± 0.89	31.83 (0.58)	4900.00	10.21	<0.001 *	0.86	[22.41–23.12]
		Negative	70	9.30 ± 2.33	9.08 (2.38)					
	GSH	Positive	70	32.92 ± 3.25	33.37 (2.39)	0.00	−10.21	<0.001 *	0.86	[−70.60–−69.40]
		Negative	70	103.12 ± 1.63	103.30 (1.86)					
*Campylobacter* (Sheep)	MDA	Positive	66	32.14 ± 0.90	31.82 (0.58)	3300.00	9.20	<0.001 *	0.85	[22.43–23.23]
		Negative	50	9.22 ± 2.57	8.99 (2.22)					
	GSH	Positive	66	32.86 ± 3.30	33.37 (2.39)	0.00	−9.20	<0.001 *	0.85	[−70.57–−69.22]
		Negative	50	103.14 ± 1.44	103.22 (1.86)					
*Campylobacter* (Goat)	MDA	Positive	4	32.10 ± 0.64	31.89 (0.45)	80.00	3.10	0.002	0.63	[21.57–23.88]
		Negative	20	9.51 ± 1.63	9.25 (1.68)					
	GSH	Positive	4	33.83 ± 2.39	33.01 (2.41)	0.00	−3.10	0.002	0.63	[−71.45–−66.07]
		Negative	20	103.05 ± 2.07	103.33 (1.06)					
Intra-Positive Group Species	MDA	Sheep	66	32.14 ± 0.90	31.82 (0.58)	123.50	−0.22	0.839	0.03	[−1.22–0.25]
		Goat	4	32.10 ± 0.64	31.89 (0.45)					
	GSH	Sheep	66	32.86 ± 3.30	33.37 (2.39)	125.50	−0.16	0.879	0.02	[−3.86–1.49]
		Goat	4	33.83 ± 2.39	33.01 (2.41)					
Intra-Negative Group Species	MDA	Sheep	50	9.22 ± 2.57	8.99 (2.22)	426.00	−0.96	0.339	0.11	[−1.42–0.37]
		Goat	20	9.51 ± 1.63	9.25 (1.68)					
	GSH	Sheep	50	103.14 ± 1.44	103.22 (1.86)	486.50	−0.18	0.866	0.02	[−0.64–0.39]
		Goat	20	103.05 ± 2.07	103.33 (1.06)					

Note: * *p* < 0.001; n: sample size; SD: standard deviation; Median (IQR): median (interquartile range, Q3–Q1); U: Mann–Whitney U test statistic; Z: standardized Z-score; *p*: statistical significance value (α = 0.05). Effect Size (r): calculated as the non-parametric effect size coefficient (r = |Z|/√N). Grading: r < 0.10 (negligible), 0.10 ≤ r < 0.30 (small), 0.30 ≤ r < 0.50 (medium), and r ≥ 0.50 (large). 95% CI: 95% confidence interval for the difference between medians calculated using the 5000-resample Bootstrap method.

**Table 3:** Spearman rank correlation coefficients (ρ), *p*-values, and inter-group evaluations between serum MDA and GSH levels in aborted small ruminants.

Category/Group	Sample (*n*)	Spearman’s Coefficient (ρ)	*p*-Value
Total Sample (Overall)	140	−0.736	<0.001
*Campylobacter* spp. Positive Group	70	0.011	0.929
*Campylobacter* spp. Negative Group	70	0.106	0.383
Sheep Species (Ovine)	116	−0.738	<0.001
Goat Species (Caprine)	24	−0.334	0.111

n: sample size; ρ: Spearman’s rank correlation coefficient; *p*: statistical significance value (α = 0.05). Correlation grading: |ρ| < 0.30 (weak), 0.30 ≤ |ρ| < 0.70 (moderate/strong), and |ρ| ≥ 0.70 (very strong). Although an extremely strong negative correlation was detected at the overall sample level (ρ = −0.736), no significant correlation was found between MDA and GSH within the *Campylobacter* spp.-positive and pathogen-negative groups themselves (*p* > 0.05). This confirms that the infection induces spatial clinical clustering between cohorts rather than a continuous intra-group trend.

## Data Availability

The original contributions presented in this study are included in the article. Further inquiries can be directed to the corresponding author.

## Abbreviations

The following abbreviations are used in this manuscript:

MDA	Malondialdehyde
GSH	Glutathione
PCR	Polymerase Chain Reaction
*C. burnetii*	*Coxiella burnetii*
*C. abortus*	*Chlamydia abortus*
SOD	Superoxide Dismutase
SD	Standard Deviation
TAS/TOS	Total Antioxidant Status/Total Oxidant Status
